# Environmental exposure assessment of lead and cadmium in street vended foods sold in selected locations in Kenya

**DOI:** 10.1002/fsn3.3344

**Published:** 2023-03-28

**Authors:** Johnson Mwove, Samuel Imathiu, Irene Orina, Paul Karanja

**Affiliations:** ^1^ Department of Human Nutrition Sciences, School of Food and Nutrition Sciences Jomo Kenyatta University of Agriculture and Technology Nairobi Kenya; ^2^ Department of Food Technology Chuka University Chuka Kenya; ^3^ Department of Food Science and Technology, School of Food and Nutrition Sciences Jomo Kenyatta University of Agriculture and Technology Nairobi Kenya

**Keywords:** food safety, heavy metal exposure assessment, human health, lead and cadmium, street vended foods

## Abstract

The preparation and handling practices, as well as raw materials for street food vending businesses, could be sources of toxic heavy metals in street vended foods (SVFs). The objective of this study was to assess the levels of lead (Pb) and cadmium (Cd) contamination in ready‐to‐eat SVFs sold in selected locations within Thika town, Kenya. A total of 199 samples consisting of cereal‐based foods, sliced fruits, salads, groundnuts, tubers, fresh fruit juices, eggs, smokies, and sausages were randomly collected for analysis. The concentration of Pb and Cd in street vended foods (SVFs) was determined by atomic absorption spectrophotometry. The results indicated that at least one of the food sample types was contaminated with Pb. The level of Pb contamination in SVFs ranged between 0.271 ± 0.070 and 1.891 ± 0.130 mg/kg with groundnuts recording significantly (*p* < .0001) higher levels (1.891 mg/kg) than all other food samples. Cadmium contamination levels in the SVF samples ranged between 0.001 ± 0.001 and 0.010 ± 0.003 mg/kg. Significantly (*p* < .0001) high levels of Cd were observed in cereal‐based foods (0.010 mg/kg) and fresh fruit juices (0.008 mg/kg). The Pb concentrations reported in this study are a food safety concern since they exceed the maximum recommended limits set by the Joint Food and Agriculture Organization (FAO)/World Health Organization (WHO) food standards program. There is therefore a need for the establishment and enforcement of policies to govern the street food vending businesses to reduce heavy metal contamination in the SVFs.

## Graphical abstract

Heavy metals, such as lead and cadmium are extremely toxic to human health and capable of bioaccumulation. Continuous ingestion of these heavy metals has a damaging effect on human health. The presence of lead and cadmium in street vended foods therefore poses a public health concern considering the huge population of people who consume these foods.

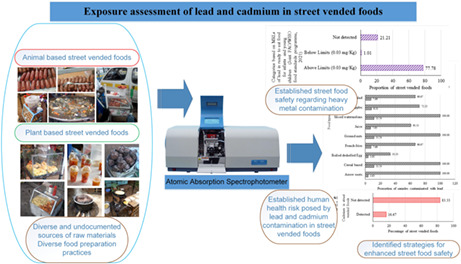

## 1 INTRODUCTION

The vast growing urban population in developing countries such as Kenya has stimulated the demand for affordable and readily accessible ready‐to‐eat (RTE) meals. This has, in turn, fueled the street vending business which offers RTE foods and beverages that are cheap, convenient, and accessible to a vast number of low‐income consumers (Imathiu, 2017; Proietti et al., 2014). In addition, street food plays an important socioeconomic role by offering jobs and income for those involved in their preparation and selling thus empowering the local economy in developing countries and improving livelihoods (Proietti et al., 2014; Verma et al., 2023).

Besides these numerous advantages offered by street foods, safety concerns have been raised regarding contamination with microbial or chemical toxic elements (Ankar‐Brewoo et al., 2020; Proietti et al., 2014; Verma et al., 2023). Heavy metals are among the chemical contaminants that may potentially contaminate street vended foods (SVFs). Contamination may occur at any step from the production of raw materials to street food preparation and eventual vending (Proietti et al., 2014). It has been reported that the use of unsuitable cookware, utensils, and food packaging material could release harmful chemicals such as heavy metals into the food (Letuka et al., 2023; Pereira et al., 2022). In addition, the source of raw food products and exposure to environmental contaminants, such as vehicle exhaust emissions and airborne dust particles may cause contamination of food with heavy metals (Ali & Al‐Qahtani, 2012). For instance, the vegetables used by street food vendors may be contaminated with significant levels of heavy metals due to farming on contaminated soils, irrigation with polluted wastewater, as well as industrial and transportation emissions that are released into the environment (Ankar‐Brewoo et al., 2020; Fasinu & Orisakwe, 2013; Iriabije & Uwadiae, 2020; Osaili et al., 2016). The growing of vegetables in cities and most industrial places has been reported to yield foodstuff contaminated with heavy metals (Ali & Al‐Qahtani, 2012). Njagi et al. (2017) reported that vegetables grown around dumpsites in Nairobi city County in Kenya were heavily contaminated with mercury (Hg) and lead (Pb). Foods of animal origin are also at risk of contamination with heavy metals due to exposure during the feeding of the animals, transportation, and handling practices at the retailing stages (Ankar‐Brewoo et al., 2020).

Several researchers have reported the presence of heavy metals including Pb and Hg in SVFs (Badr & Arafa, 2023; Chavez et al., 2014; Ekhator et al., 2017; Ihsan & Edwin, 2018; Mohammed, 2010). In a study carried out in Sudan, Pb was found in various food products including ice cream and peanut butter (Mohammed, 2010). Lead, cadmium (Cd), and Hg were also reported in vegetables, cereals, and fruits sold in Saudi Arabian markets (Ali & Al‐Qahtani, 2012). In Nigeria, Ogunkunle et al. (2014) reported the presence of Pb, Cd, copper, nickel, and cobalt in street vended mangoes, pawpaw, and watermelons while Ekhator et al. (2017) reported the presence of Pb, Hg, antimony, manganese, aluminum, and Cd in SVFs in Nigeria. In Kenya, research on heavy metals especially RTE food products is limited. Kinyanjui (2009) found Pb levels of up to 0.38 mg/100 g in fruits and vegetables sold in Kisumu, Kenya.

Heavy metals are extremely toxic to human health and are capable of bioaccumulation (Ismail et al., 2017; Khan et al., 2015; Letuka et al., 2023). Continuous ingestion of heavy metals has a damaging effect on human health (Israel et al., 2020; Jaishankar et al., 2014). The adverse effects of heavy metals on human health include structural damage, renal failure, damage to cells and tissues, osteoporosis, lung or even blood cancer, hormone imbalances, gastrointestinal problems, and anemia (Ismail et al., 2017; Letuka et al., 2023). Previous research has also linked heavy metals to the increasing cases of cancer in sub‐Saharan Africa (Fasinu & Orisakwe, 2013). Problems caused by these toxic contaminants may not be immediate but accumulation in the body may reach toxic levels causing health issues later in life (Ogu & Akinnibosun, 2020). For SVFs that have minimal regulation, research on heavy metal contamination can inform the making of policies to govern street food quality. However, there is little knowledge of dangerous chemical pollutants such as levels of heavy metals in the SVFs in Kenya. Knowledge regarding concentrations of heavy metals in foods and their dietary intake is very useful in the determination of their risk to human health (Ankar‐Brewoo et al., 2020; Khan et al., 2015). Therefore, this study aimed to determine the level of selected heavy metals in SVFs sold in Thika town, Kenya.

## 2 MATERIALS AND METHODS

### 2.1 Study site

**FIGURE 1:**
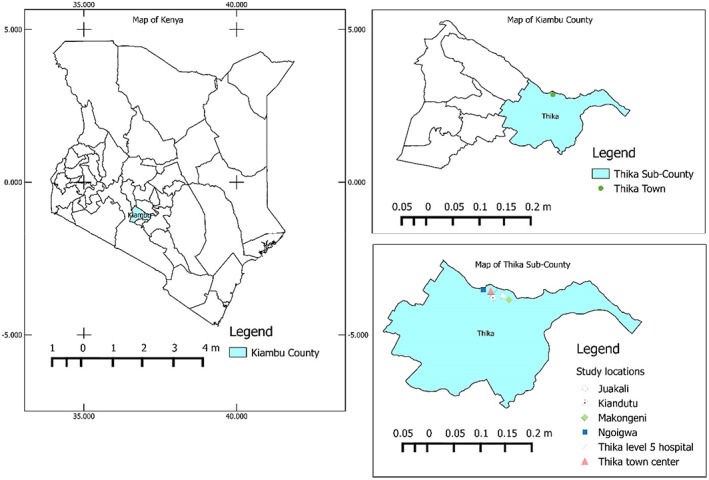
Map of the study area showing the study locations in Thika town.

This study was carried out in six locations including Thika town center, Goingwa, Juakali area, Makongeni, Kiandutu, and Thika Level 5 hospital area in Thika town, Kenya as shown in Figure 1. Heavy metal analysis was carried out at Chuka University, Department of Physical Sciences, Chemistry laboratory.

### 2.2 Sampling of street vended foods

The sampling of street vended food samples was done from randomly selected street food vendors in each study location. There were 12 ready‐to‐eat food sample types on offer by street food vendors which were selected for analysis. The food samples were categorized as either food of animal origin (sausages, smokies, and eggs) or foods of plant origin (cereal‐based foods, french fries, groundnuts, fresh fruit juices, sliced watermelons, sliced pineapples, salads, arrowroots, and sweet potatoes). Approximately, 200–300 g of each food sample was collected from the randomly selected street food vendors and bagged in a sterile 500 g sample bag. Samples were immediately transferred into a cooler box (4°C) for transportation to the laboratory for analysis.

### 2.3 Preparation of reagents and standard curves

All reagents used were of analytical grade. Analytical standards for Pb and Cd were procured from Sigma Aldrich Company. The standard analytes for the calibration curve were made by diluting a 1000 mg/L stock solution of the examined element. For each of the elements to be examined, different concentrations were prepared from the stock solution and a calibration curve was plotted using concentration and absorbance data from each set of standards.

### 2.4 Sample preparation, digestion, and analysis

The presence of Pb and Cd in food samples was determined using atomic absorption spectrophotometer (PG 990 model) as described by Puwastien et al. (2011). Solid samples were dried at 105°C for 24 h and homogenized in a laboratory grinder. Fresh fruit juices, sliced watermelons, sliced pineapples, and salads were blended into a homogenous mixture and used in their liquid form for analysis. Dry ashing was done in a muffle furnace at 525°C for 8 h using 3 g of the dried samples and 10 g of the fresh fruit juices, sliced watermelons, sliced pineapples, and salads. The fresh fruit juices, sliced watermelons, sliced pineapples, and salad samples were first dried in an oven at 105°C until all moisture was removed before charring and ashing. The ash obtained was dissolved in 1 N Nitric acid solution, filtered using the Whatman filter paper No. 1, and the volume adjusted to 50.0 mL with distilled water. A blank sample was prepared in the same way. The instrument parameters were as follows: wavelengths were 283.3 and 228.8 nm, for Pb and Cd, respectively. In both cases, the lamp current was 2.0 A while the slit was 0.4 nm. The limit of detection (LOD) and the limit of quantification (LOQ) were determined using blank determination as described by Shrivastava and Gupta (2011). The LOD was 0.062 and 0.001 while the LOQ was 0.064 and 0.004 for Pb and Cd, respectively. Measurements were carried out in an air/acetylene flame.

### 2.5 Data analysis

All analyzed sample results were subjected to statistical analysis using SAS (Version 9.4) software for analysis of variance (ANOVA) and mean separation using Tukey's honestly significant difference (HSD) test at 95% confidence interval.

## 3 RESULTS AND DISCUSSION

### 3.1 Exposure assessment of cadmium in street vended foods

Cadmium (Cd) contamination was only quantified in cereal‐based foods, eggs, french fries, fresh fruit juices, sliced watermelons, sausages as well as sweet potato samples (Table 1). Detectable contamination levels in all street vended foods (SVFs) ranged between 0.001 ± 0.001 and 0.010 ± 0.003 mg/kg. Significantly (*p* < .0001) higher levels of Cd were observed in cereals (0.010 mg/kg) and fresh fruit juices (0.008 mg/kg) as compared with all other samples. The lowest detectable levels of Cd were observed in french fries although this was not significantly different from the sliced watermelons. Cereal‐based foods had the highest number of samples contaminated with Cd which were only found in three study locations including Kiandutu, Ngoigwa and Thika town center (Table 2). In contrast to this study, Ezeilo et al. (2020) reported higher contamination levels ranging between 0.03 and 0.70 mg/kg in fruits and vegetables. Hassan et al. (2022) reported levels of cadmium between 0.001 and 1.000 mg/kg in street vended foods (*shawarma*, *fruit chaat*, and *dahi baray*) sold in Faisalabad city, Pakistan. Jin et al. (2023) also reported cereal‐based foods and aquatic foods to be the main contributors of Cd among other heavy metals in the diet. The high levels of Cd in cereals and fresh fruit juices in this study could have originated from contaminated raw materials that were used in the preparation of the SVFs. Cadmium contamination reaching the food chain may originate from geogenic and anthropogenic activities (Bolan et al., 2013). In other studies, the presence of Cd in plant‐based foods, such as cereals and fruits has been attributed to Cd impurities in fertilizers and amendments applied to soils (McLaughlin & Singh, 1999). Furthermore, wastewater carrying industrial wastes that may be used to irrigate agricultural lands, the burning of fossil fuels such as coal or oil, and the incineration of municipal waste may be a major source of Cd in the food chain (Adedapo & Adeoye, 2014; Mahmood et al., 2019). Plastic utensils used for handling food have also been found to contaminate food with Cd (Pereira et al., 2022). Plastic utensils are common in street vending environments in the study area as reported earlier (Mwove et al., 2020). Different foods undergo different handling and preparation practices. These practices influence the presence of heavy metals in foods in different ways. This may explain the differences observed in the concentration of cadmium in different food samples.

**TABLE 1:** The level of lead and cadmium in specific street vended foods sold in selected locations in Thika town, Kenya.

Sample	Number of samples	Cadmium (mg/kg)	MRL for Cd	Lead (mg/kg)	MRL for Pb
Arrowroots	6	<d	0.1	0.558 ± 0.093^b^	0.1
Cereal‐based	18	**0.010 ± 0.003** ^**a**^	0.1**	0.580 ± 0.057^b^	0.1–0.2^++++^
Boiled deshelled eggs	18	0.004 ± 0.002^b^		0.442 ± 0.204^c^	0.1*
French fries	18	**0.001 ± 0.001** ^**cd**^	0.1	0.511 ± 0.149^bc^	0.1
Groundnuts	18	<d		1.891 ± 0.130^a^	‐
Juice	18	0.008 ± 0.003^a^		0.271 ± 0.071^e^	0.03
Watermelon	18	0.003 ± 0.001^bc^		0.466 ± 0.081^c^	0.1
Pineapple	18	<d		0.335 ± 0.059^de^	0.1
Salad	18	<d	0.05^+++^	0.547 ± 0.200^b^	0.05–0.1^+^
Sausages	18	0.005 ± 0.003^b^		0.560 ± 0.073^b^	0.1^++^
Smokies	18	<d		0.577 ± 0.086^b^	0.1^++^
Sweet potatoes	12	**0.001 ± 0.000** ^**d**^	0.1	0.356 ± 0.100^d^	0.1

*Note*: Data presented as mean ± standard deviation. Values in each column with different superscripts are significantly different (*p* < .05). <d means below the limit of detection (LOD) = 0.001. MRL is Maximum Recommended Limit. *Level suggested by Joint FAO/WHO food standards program (2021). ^+^Cd range given for fruiting vegetables (0.05) and bulb vegetables (0.1), ^+++^Pb level in fruiting vegetables (0.05) and bulb vegetables (0.05), ^++^Pb level set for meat from poultry, cattle, pigs and sheep, ^++++^Pb range taken for pulses (0.1) and cereal grains (0.2), **Cd Level for pulses (0.1) and cereal grains (0.1) (Codex Alimentarius Commission, 2019). The means in bold are below the MRLs specified.

**TABLE 2:** Cadmium contamination levels (mg/kg) in different street vended food samples within different study locations in Thika town, Kenya.

Food type	Study areas						Limit
	Hospital area	Juakali area	Kiandutu	Makongeni	Ngoigwa	Thika town center	
Arrow roots	‐	‐	‐	<d	<d	‐	0.1
Cereal‐based	<d	<d	**0.025** **±** **0.003** ^a^	<d	**0.006** **±** **0.001** ^**b**^	**0.03** **±** **0.002** ^**a**^	0.1**
Boiled deshelled eggs	<d	<d	<d	<d	0.022 ± 0.005^a^	<d	‐
French fries	<d	<d	<d	**0.006** **±** **0.004** ^**a**^	<d	<d	0.1
Groundnuts	<d	<d	<d	<d	<d	<d	‐
Juice	0.036 ± 0.001^a^	<d	<d	<d	0.01 ± 0.002^b^	<d	‐
Mellon	0.013 ± 0.002^a^	<d	<d	0.006 ± 0.001^b^	<d	<d	‐
Pineapple	<d	<d	<d	<d	<d	0.002 ± 0.001^a^	‐
Salad	<d	<d	<d	<d	<d	<d	0.05*
Sausages	<d	<d	<d	<d	<d	0.029 ± 0.003^a^	‐
Smokies	<d	<d	<d	<d	<d	<d	‐
Sweet potatoes	‐	<d	‐	<d	<d	**0.002** ** ±** **0.001** ^a^	0.1

*Note*: Data presented as mean ± standard deviation. Values in each row with different superscripts are significantly different (*p* < .05). <d means below the limit of detection (LOD) = 0.001. *Level of Cd in fruiting vegetables (0.05) and bulb vegetables (0.05), **Level of Cd for pulses (0.1) and cereal grains (0.1), (Codex Alimentarius Commission, 2019). MRL is Maximum Recommended Limit. The means in bold are below the MRLs specified.

**FIGURE 2:**
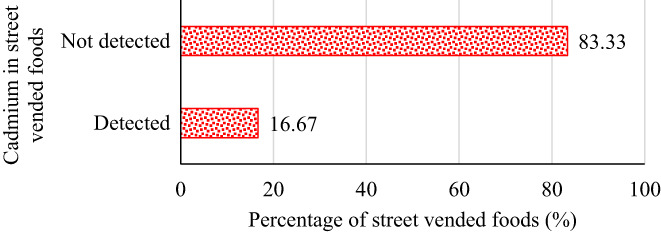
The proportion of street vended foods contaminated with cadmium in Thika town, Kenya.

Although Cd was detected in 16.67% of street vended food samples in this study (Figure 2), none of the samples had contamination levels above MRLs established by Codex Alimentarius Commission (2019) for cereal‐based products, french fries, and sweet potatoes.

The level of Cd was highest in SVFs sold around the hospital area (0.005 mg/kg) and Thika town center (0.006 mg/kg) as shown in Table 3. As earlier observed, Thika town center also recorded the highest number of samples contaminated with Cd. In addition, at least one foodstuff was contaminated with Cd in all study locations except the Juakali area (Table 2). The high levels of contamination of foods with Cd in areas around the hospital area and Thika town center could be attributed to the heavy traffic around these places as compared with the Juakali area. Exhaust and non‐exhaust vehicle emissions in urban and motorway road dust have been found to contain Cd, among other heavy metals which may contaminate food and the environment (Adamiec et al., 2016; Chenery et al., 2020; Ferretti et al., 1995). Thus, foods that are not covered adequately during preparation or sold along roads that have heavy traffic may be contaminated with Cd to a larger extent as compared with those sold further away. Furthermore, water used for handling food has been reported to be a potential source of Cd in food (Musa et al., 2020). The presence of Cd in these SVFs poses a health risk to consumers, especially the sick people visiting Thika Level 5 hospital when they consume these foods.

**TABLE 3:** The level of lead and cadmium in street vended foods within selected locations in Thika town, Kenya.

Location	Number of samples	Cd (mg/kg)	Pb (mg/kg)
Hospital Area	30	0.005 ± 0.002^a^	0.687 ± 0.136^a^
Juakali Area	33	<d	0.436 ± 0.060^c^
Kiandutu	30	0.002 ± 0.001^b^	0.554 ± 0.092^b^
Makongeni	36	0.001 ± 0.000^c^	0.410 ± 0.094^c^
Ngoigwa	36	0.003 ± 0.001^b^	0.935 ± 0.137^a^
Thika town center	33	0.006 ± 0.002^a^	0.570 ± 0.116^b^

*Note*: Data presented as mean ± standard deviation. Values in each column with different superscripts are significantly different (*p* < .05). <d means below the limit of detection (LOD) = 0.001.

Although the levels reported in this study were below the MRLs established by Codex Alimentarius Commission (2019), the presence of Cd in foods is a major health concern since there is no treatment for Cd poisoning (Ellen & Costa, 2010). Cadmium has been classified by the International Agency for Research on Cancer (IARC) as carcinogenic to humans (Group 1), with sufficient evidence for lung cancer and limited evidence for kidney, liver, and prostate cancer (Joint FAO/WHO Expert Committee on Food Additives, 2011). Excessive intake and long‐term exposure to Cd have also been reported to cause serious illnesses such as *itai‐itai* disease, chemical pneumonitis, and chronic obstructive lung disease (Bolan et al., 2013; Ellen & Costa, 2010; Letuka et al., 2023; Rahimzadeh et al., 2017). Therefore, its presence in SVFs poses a public health concern considering the huge population of people who frequently consume these foods daily.

### 3.2 Exposure assessment of lead in the street vended foods

**FIGURE 3:**
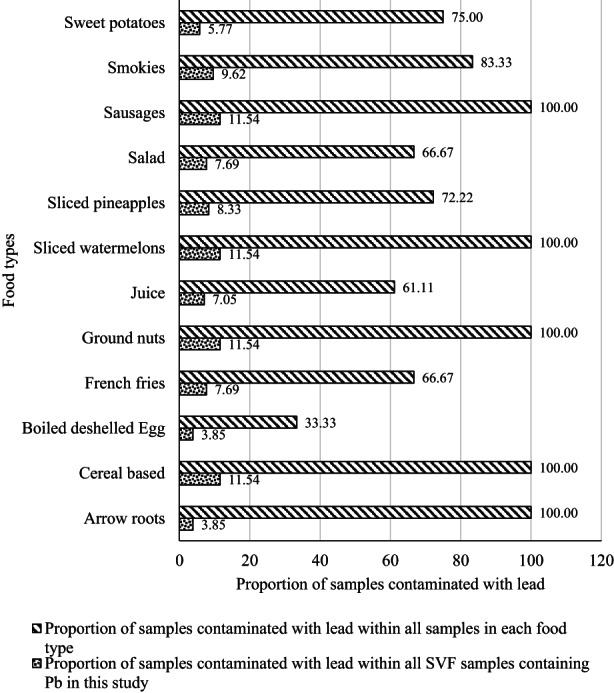
The proportion of street vended food samples contaminated with Pb within each food type.

At least one of all food sample types was contaminated with Pb (Table 1). The level of Pb contamination quantified in street vended food samples ranged between 0.271 ± 0.07 and 1.891 ± 0.130 mg/kg. The highest contamination levels were found in groundnuts (1.891 mg/kg) while the lowest contamination was found in fresh fruit juices at 0.271 mg/kg. All arrowroots, cereal‐based foods, groundnuts, sliced watermelons, and sausage samples collected were contaminated with Pb (Figure 3). Boiled deshelled eggs were the least contaminated with only 33.33% of all the egg samples contaminated with Pb.

In contrast to this study, Ezeilo et al. (2020) reported higher Pb contamination levels ranging between 1.23 ± 0.01 and 10.66 ± 0.01 mg/kg in fruits (banana (*Musa paradisiaca*), pawpaw (*Carica papaya*), watermelon (*Citrulus laratus*), apple (*Malus domestica*), cucumber (*Cucumis sativus*), guava (*Psidium guajava*)), bush mango (*Irvingia gabonensis*) and vegetables (bitter leaf (*Vernonia amygdalina*), pumpkin leaf (*Telfairia occidentalis*), uziza leaf (*Piper guineese*), scent leaf (*Ocimum gratissimum*), water leaf (*Talinum triangulare*) and oha leaf (*Pterocarpa mildraedil*)) in Anambra State, Nigeria. Similarly, Ankar‐Brewoo et al. (2020) reported higher Pb levels ranging from 0.9 to 18 mg/kg in fried rice and chicken samples in Kumasi, Ghana. Contamination levels quantified in sliced watermelons and sliced pineapples were higher than those reported by Rotimi et al. (2018) who found pineapples and watermelons to contain 0.10 and 0.13 mg/kg, respectively in a study investigating heavy metal contamination in fruits commonly sold from selected markets in Lagos, Nigeria.

**FIGURE 4:**
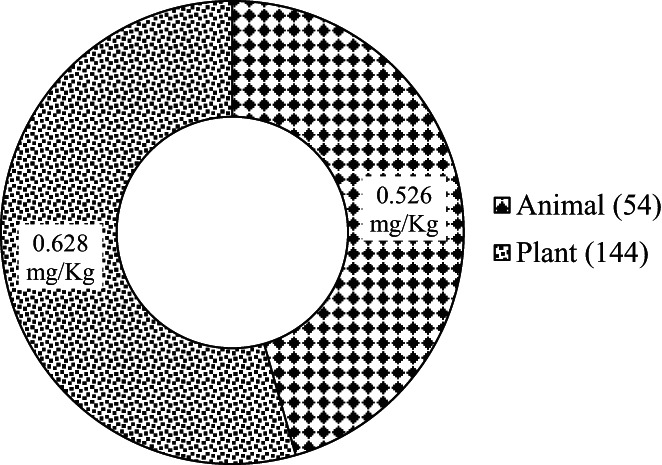
Mean lead contamination levels in animal and plant‐based street vended food products in Thika town, Kenya. The number of samples is indicated in brackets.

Plant‐based foods had significantly higher Pb contamination levels compared with animal‐based foods as shown in Figure 4. The sources of Pb in the environment are both natural and anthropogenic (Davis et al., 2009). The contamination in foods may originate from vehicle exhaust emissions and contaminated soils which in turn contaminate plant‐based foods and feeds, with the latter contaminating animal‐based food products when animals consume the contaminated feeds (FDA, 2020). Letuka et al. (2023) reported that some unhygienic handling practices that may cause cross‐contamination, food preparation, and storage materials as well as the type of fuel used for cooking may affect the contamination levels of Pb in food. In addition, Musa et al. (2020) reported the presence of Pb (0.21 ± 0.017 mg/kg) and Cd (0.015 ± 0.003 mg/kg) in water used by food providers. Thus, the water used in this study could have served as a source of Pb contamination. The high contamination in plant‐based foods especially groundnuts may be attributed to the contamination of soil with Pb in the areas where these foods were grown. Researchers have reported widespread contamination of soil, water, and food in Kenya. In a study carried out in Kisumu, Kenya, Makokha et al. (2008) reported widespread contamination of lead in soil, drinking water, vegetables, and fish. In addition, industrial pollution with heavy metals has also been reported. For instance, Kinuthia et al. (2020) while studying the levels of heavy metals in wastewater soil samples collected from open drainage channels in Nairobi, Kenya reported Pb levels in wastewater above WHO, US EPA, and Kenya allowable limits. Furthermore, the soil was also contaminated with Pb among other heavy metals above WHO limits for agricultural soils.

These contaminated discharges are released into the waterways further resulting in the contamination of water and irrigated lands. In a study carried out to determine the levels of heavy metals in sediments of the Masinga reservoir in Kenya, Nzeve et al. (2018) reported the presence of Pb at levels lower than the WHO set limit. Thus, street vended foods could have been prepared from contaminated raw materials or water, or exposed to contaminated dust when left uncovered during sale in areas that have heavy traffic. Leaching from handling and storage containers may also explain the presence of lead in all the food types examined.

**FIGURE 5:**
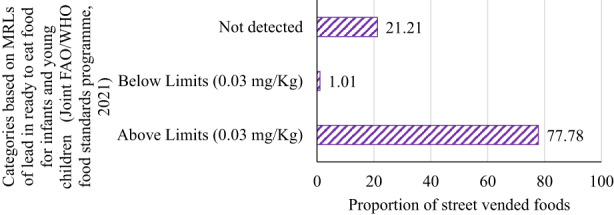
Lead contamination levels in street vended foods compared with the limits suggested by the Joint FAO/WHO Food Standards Program (2021) for RTE foods for infants and young children.

On average, all of the street vended food samples were above the MRLs established by the Codex Alimentarius Commission (2019) and above the MRL (0.03 mg/kg) suggested by the Joint FAO/WHO Food Standards Program (2021) for RTE foods intended for infants and young children regarding Pb contamination. As shown in Figure 5, 78.79% of all food samples had Pb within detectable levels of which 77.78% had Pb levels above the MRL suggested by the Joint FAO/WHO Food Standards Program (2021) for RTE foods intended for infants and young children. This poses serious public health concerns considering that children are among the vast number of street food consumers.

Lead contamination was highest around the hospital area (0.687 mg/kg) and Ngoigwa (0.935 mg/kg) as shown in Table 3. Juakali area (0.436 mg/kg) and Makongeni area (0.410 mg/kg) recorded the lowest contamination levels which were significantly (*p* < .0001) lower than all other study areas. At least one street vended food sample in each food type was reported to contain Pb in all study locations (Table 4). Eggs were the least contaminated food type with contamination only reported around the hospital area and Kiandutu area. SVFs were most contaminated with Pb in the Ngoigwa area, with arrowroots, french fries, groundnuts, sliced pineapples, salad, smokies, and sweet potatoes showing the highest contamination levels. Atter et al. (2015) reported Pb contamination levels averaging between 0.34 and 1.25 mg/kg in a street vended traditional maize beverage sold in Ghana. Ekhator et al. (2017) reported Pb contamination levels ranging between 0.014 and 1.37 mg/kg in SVFs consumed in Mid‐West Nigeria. Hassan et al. (2022) reported the levels of lead between 0.014 and 0.955 mg/kg in street vended foods (shawarma, fruit chaat, and dahi baray) sold in Faisalabad city, Pakistan.

**TABLE 4:** Lead contamination levels (mg/kg) in different street vended food samples within different study locations in Thika town, Kenya.

Food type	Study area						Limits
	Hospital area	Juakali area	Kiandutu	Makongeni	Ngoigwa	Thika town center	
Arrow roots	‐	‐	‐	0.354 ± 0.011^b^	0.762 ± 0.04^a^	‐	0.1
Cereal‐based	0.423 ± 0.039^c^	0.644 ± 0.012^b^	0.962 ± 0.017^a^	0.202 ± 0.007^d^	0.635 ± 0.026^b^	0.616 ± 0.046^b^	0.1–0.2^+++^
Boiled deshelled eggs	2.302 ± 0.058^a^	<d	0.348 ± 0.009^b^	<d	<d	<d	0.1*
French fries	0.182 ± 0.058^b^	0.14 ± 0.037^b^	<d	<d	1.402 ± 0.041^a^	1.342 ± 0.049^a^	0.1
Ground nuts	1.766 ± 0.087^c^	1.026 ± 0.048^d^	1.674 ± 0.081^c^	1.928 ± 0.08b^c^	2.75 ± 0.118^a^	2.205 ± 0.074^b^	‐
Juice	0.77 ± 0.023^a^	0.462 ± 0.059^b^	0.373 ± 0.006^b^	0.019 ± 0.014^c^	<d	<d	0.03
Sliced watermelons	0.321 ± 0.024^c^	0.128 ± 0.02^d^	0.598 ± 0.004^b^	1.118 ± 0.039^a^	0.446 ± 0.042^c^	0.186 ± 0.02^d^	0.1
Sliced pineapple	<d	0.38 ± 0.039^b^	0.008 ± 0.008^c^	0.511 ± 0.004^a^	0.567 ± 0.01^a^	0.545 ± 0.002^a^	0.1
Salad	0.22 ± 0.043^c^	<d	0.221 ± 0.01^c^	<d	2.347 ± 0.079^a^	0.497 ± 0.005^b^	0.05–.1^+^
Sausages	0.061 ± 0.007^c^	0.92 ± 0.03^a^	0.354 ± 0.027^c^	0.467 ± 0.026^c^	0.727 ± 0.034^b^	0.831 ± 0.048^ab^	0.1^++^
Smokies	0.825 ± 0.046^a^	0.455 ± 0.05^b^	1 ± 0.003^a^	0.323 ± 0.015^b^	0.857 ± 0.073^a^	<d	0.1^++^
Sweet potatoes	‐	0.638 ± 0.018^b^	‐	<d	0.734 ± 0.014^a^	0.053 ± 0.011^c^	0.1

*Note*: Data presented as mean ± standard deviation. Values in each row with different superscripts are significantly different (*p* < .05). <d means below the limit of detection (LOD) = 0.062. *Level suggested by Joint FAO/WHO Food Standards Program (2021). ^+^ Range given for fruiting vegetables (0.05) and bulb vegetables (0.1), ^++^ Level set for meat from poultry, cattle, pigs and sheep, ^+++^ Range taken for pulses (0.1) and cereal grains (0.2), (Codex Alimentarius Commission, 2019).

These levels are high and comparable to the contamination levels reported in this study. This shows that Pb is a persistent contaminant in the environment in many places around the world. The high levels of contamination of foods with Pb in areas around the hospital and Ngoigwa could be attributed to the heavy traffic around these places. Exhaust and non‐exhaust vehicle emissions in urban and motorway road dust have been found to contain Pb, which may contaminate food and the environment (Adamiec et al., 2016; Ferretti et al., 1995). The low contamination reported in eggs could be because eggs were deshelled before sampling was carried out. Eggshells have been reported to be an avenue for the excretion of lead in birds (Burger, 1994; Trampel et al., 2003). Thus, contamination with Pb that was possibly in the shell may have been removed when the eggs were deshelled.

Like many other heavy metals, Pb is not biodegradable and thus it does not disappear from the environment over time (FDA, 2020). Furthermore, Pb builds up in the body over time, and thus, even low‐level chronic exposure can be dangerous (FDA, 2020; Letuka et al., 2023; Naranjo et al., 2020). Infants, young children, pregnant women and their fetuses, and individuals with chronic health issues are particularly vulnerable to Pb poisoning (FDA, 2020). According to Dobaradaran et al. (2010), Pb is toxic even at trace levels. Lead may cause renal failure, liver damage, impaired hearing, mental retardation, and shortened gestation in humans (Ankar‐Brewoo et al., 2020; Islam et al., 2014). In children, it can cause adverse and permanent neurodevelopmental problems resulting in learning deficits, behavioral problems, and a lower IQ in early childhood (FDA, 2020; Naranjo et al., 2020). Thus, Pb presence in SVFs poses a public health concern considering the huge population of people who consume street vended foods.

## 4 CONCLUSION

The Pb concentrations reported in this study are a cause for concern since the vast majority of samples analyzed had contamination levels exceeding the MRLs suggested by the Joint FAO/WHO food standards program. At least one of all food sample types was contaminated with Pb, with the highest contamination levels being observed in groundnuts. In addition, at least one foodstuff was contaminated with Cd in all study locations except the Juakali area. The presence of Pb and Cd in SVFs poses a public health concern considering the huge population of people who consume these foods. Furthermore, these heavy metals can bioaccumulate in the body posing health risks later in life. Thus, there is a need for continuous monitoring of SVFs to increase awareness of heavy metal contamination in the food chain. Furthermore, the establishment and enforcement of policies to govern the SVFs businesses aimed at reducing heavy metal contamination are recommended.

## CONFLICT OF INTEREST STATEMENT

The authors declare that they do not have any conflict of interest.

## ETHICAL APPROVAL

This study does not involve any human or animal testing. Permission to undertake this study was obtained from the National Commission for Science and Technology (NACOSTI), Kenya, Research permit number: NACOSTI/P/19/87469/31129.

## Data Availability

Data available on request from the authors.
